# Bilateral differences in the trunk muscle volume of skilled golfers

**DOI:** 10.1371/journal.pone.0214752

**Published:** 2019-04-25

**Authors:** Yoka Izumoto, Toshiyuki Kurihara, Tadashi Suga, Tadao Isaka

**Affiliations:** 1 Graduate School of Sport and Health Science, Ritsumeikan University, Shiga, Japan; 2 Research Organization of Science and Technology, Ritsumeikan University, Shiga, Japan; 3 Faculty of Sport and Health Science, Ritsumeikan University, Shiga, Japan; Universite de Nantes, FRANCE

## Abstract

Repetitive mechanical load applied to a body part may lead to hypertrophy of its muscles. If a movement requires asymmetric activation of bilateral muscles, this may result in differences in muscle size between the sides. This study aimed to investigate the degree of bilateral differences in the trunk muscle volume of golfers by comparing with non-golfers. Seventeen male right-handed golfers and eleven (nine right- and two left-handed) non-golfers participated. Trunk muscle volume was determined using magnetic resonance imaging, and the degree of asymmetry was calculated as the ratio of trunk muscle volume on the left to trunk muscle volume on the right side in right-handers and vice-versa in left-handers. Golfers had significantly larger lateral abdominal wall (LA) muscle volume than non-golfers: 12.36 ± 1.12 vs. 9.96 ± 0.94 cm^3^/kg; erector spinae: 9.12 ± 1.16 vs. 7.88 ± 0.84 cm^3^/kg; psoas major (PM): 6.27 ± 0.88 vs. 5.51 ± 0.98 cm^3^/kg; rectus abdominis (RA): 4.15 ± 0.54 vs. 3.50 ± 0.64 cm^3^/kg; and multifidus: 3.61 ± 0.41 vs. 3.05 ± 0.40 cm^3^/kg (*p* < 0.05). The degree of bilateral asymmetry of the LA, PM, and RA volume was significantly greater in golfers than in non-golfers (LA: -8.63 ± 7.40% vs. 1.94 ± 2.76%; PM: -9.10 ± 5.25% vs. -0.48 ± 5.96%; RA: 6.36 ± 6.50% vs. -2.12 ± 9.64%, respectively, *p* < 0.05). Right-handed golfers had greater left LA and PM volume compared to the right (LA: 5.89 ± 0.55 vs. 6.48 ± 0.65 cm^3^/kg; PM: 3.00 ± 0.42 vs. 3.27 ± 0.47 cm^3^/kg; *p* < 0.05) and had greater right RA volume compared to the left (2.15 ± 0.32 vs. 2.00 ± 0.24 cm^3^/kg, *p* < 0.05). These findings suggest that skilled, long-term golfers develop large volume and bilateral asymmetry of their trunk muscles.

## Introduction

Athletes commonly possess greater muscle volume in specific body parts compared to non-athletes. One of the most common reasons for this is a repetitive mechanical load that is typical for the respective sport and therefore applied to these body parts over a prolonged period of time. Such adaptation could lead to the bilateral differences in trunk muscle size observed in skilled players of sports which require asymmetric trunk motion such as tennis, baseball, and cricket [1–3]. Previous studies have shown that professional tennis players have a 30% larger lateral abdominal wall muscle volume (LA; includes the external and internal abdominal oblique, and the transverse abdominal [1]), and a 58% larger rectus abdominis (RA) muscle volume than non-active people [4]. In both trunk muscles, the hypertrophy was asymmetric (18% and 35% higher volume on non-dominant side compared to dominant side LA and RA, respectively) [1, 4].

Golf is one sport that requires asymmetric trunk movement. In right-handed golfers, kinematic studies of their swing show that the trunk rotates to the left and laterally flexes to the right side while remaining in forward flexion during the downswing [5, 6]. Electromyography (EMG) studies show that the LA and RA muscles on both sides are activated separately and asymmetrically during the golf swing [7, 8] and that larger trunk rotation is associated with greater activation of the abdominal muscles [9]. The major role of the LA is to rotate the trunk [10]. Rotating the trunk to one side activates the ipsilateral internal abdominal oblique and contralateral external abdominal oblique. The major role of the RA is trunk forward flexion and also lateral flexion to the side of the actively contracting RA [10]. These results suggest that frequent golf swing practice may cause asymmetric hypertrophy of the LA and RA muscles, and skilled long-term golfers would have greater degree of bilateral difference in trunk muscles compared to non-active people who have never played the golf as well as the results of previous studies of professional tennis players [1, 4]. However, these have so far not been investigated.

The purpose of this study was to investigate the degree of bilateral differences in the trunk muscles of skilled, long-term golfers by comparing with a matched non-active subject group. It was hypothesized that the long-term practice of golf causes asymmetric hypertrophy of the trunk muscles because the golf swing exerts an asymmetric mechanical load on the trunk muscles.

## Materials and methods

### Subjects

Seventeen male right-handed golfers and eleven male non-golfers (nine right-handed and two left-handed) agreed to participate in this study (Table 1) and were assigned to two groups, golfers and non-golfers, respectively. There were no significant differences in the physical characteristics between the two groups (*p* > 0.05). The golfers had at least 10 years of experience in golf practice (12.4 ± 2.8 years), and they performed the game at a relatively high level (best score, 65.5 ± 1.8 strokes/round; average score during the last month, 74.5 ± 2.2 strokes/round). All non-golfers had never played the game and had not been involved in regular physical exercise for the last six months before the measurements. The study was approved by the Ethics Committee of Ritsumeikan University (BKC-IRB-2013-015), and written informed consent was obtained from each participant, confirming that they understood the purpose of the study and the possible risks of participating.

**Table 1:** Baseline physical characteristics of golfers and non-golfers and experience and performance level of golfers (mean ± SD).

Variables	Golfers	Non-golfers	*p*
N	17	11	
Age (years)	20.3±0.9	22.2±0.9	< 0.05
Height (cm)	171.0±5.5	171.6±4.6	n.s.
Body mass (kg)	70.2±8.9	66.5±9.9	n.s.
Golf experience (years)	12.4±2.8		
Best score (strokes / round)	65.5±1.8		
Average score (strokes / round)	74.5±2.2		

n.s., not significant

### Data collection

Magnetic resonance (MR) images of the trunk were obtained with a 1.5 tesla magnetic resonance system (Signa HDxt 1.5T; GE Healthcare UK Ltd, Buckinghamshire, England) while the subjects lay in the supine position. Serial transverse MR images (repetition time, 6700 ms; echo time, 7.2 ms; slice thickness, 10 mm; inter-spaced distance, 0 mm; field of view, 480 × 480 mm; matrix size, 512 × 512) were obtained perpendicular to the anterior abdominal wall from the first lumbar to the first sacral vertebra.

### Data analysis

**Fig 1:**
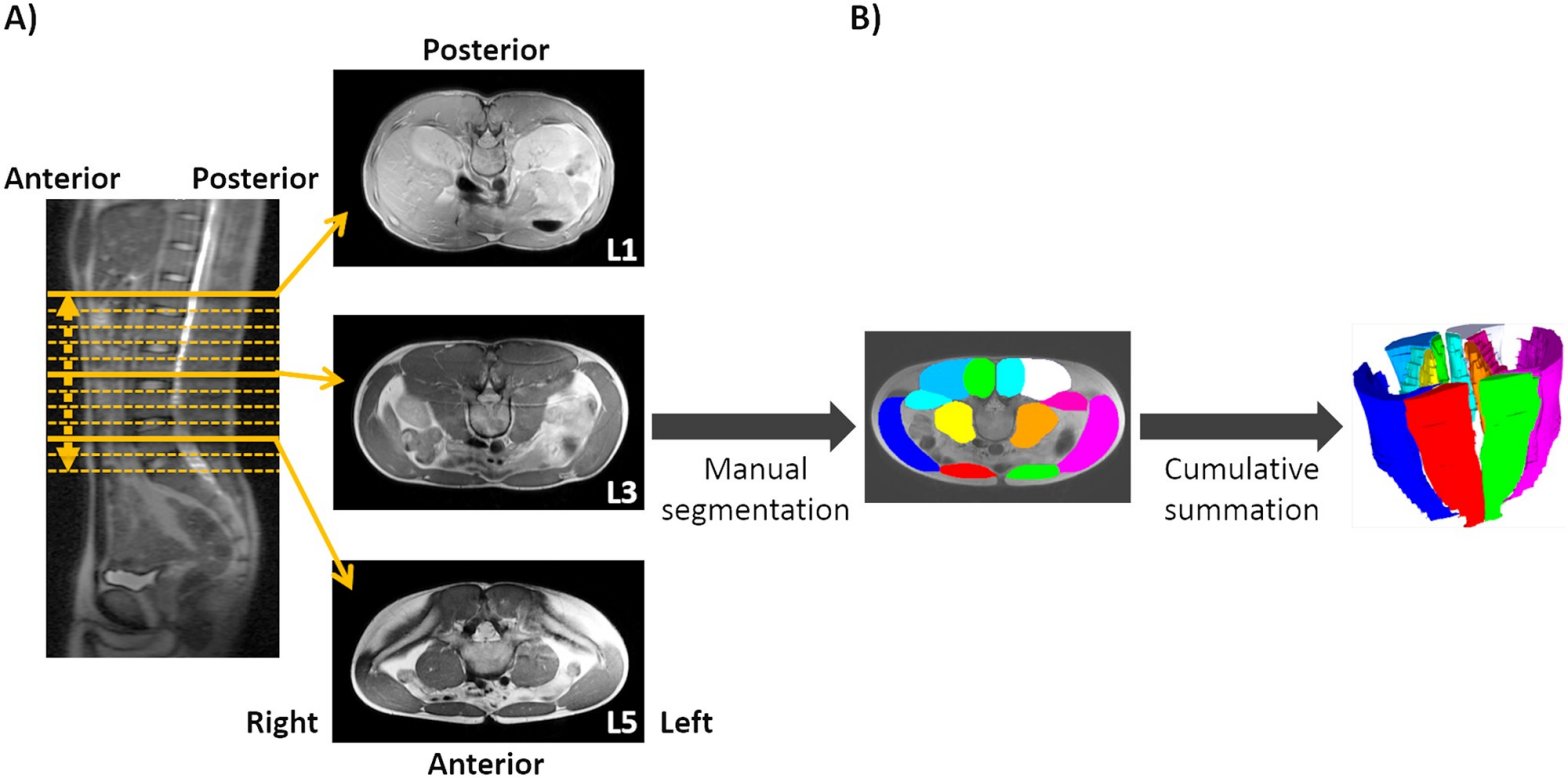
A) Transverse magnetic resonance images of the trunk; B) Manual segmentation of the trunk muscles and cumulative summation of the cross-sectional area.

MR images were used to determine the volume of both the left and right trunk muscles (Fig 1). The cross-sectional areas of the trunk muscles, i.e. the RA, LA, psoas major (PM), quadratus lumborum (QL), erector spinae (ES) and multifidus (MF), were manually determined using an image analysis software (SliceOmatic 5.0 Rev-4b2, Tomovision, Magog, Canada), and the areas of all slices were added to estimate the muscle volume of each trunk muscle. Manual segmentation of each trunk muscle was performed by a well-trained examiner. Intra-rater repeatability of manual segmentation was assessed with the use of intraclass correlations (ICCs), and the ICC (1, 2) coefficient was calculated (r = 0.98). All trunk muscle volumes were normalized for the body mass of the subject. We defined the “dominant side” of trunk muscle volume as the side of the dominant hand in both golfers and non-golfers and the non-dominant side as the opposite side. The degree of bilateral asymmetry for each trunk muscle was calculated as the ratio of the muscle volume on the non-dominant-side to- the muscle volume on the dominant side = {[muscle volume of dominant side]–[muscle volume of non-dominant side]} / [muscle volume of dominant side] × 100.

### Statistics

All results were presented as mean ± standard deviation. All trunk muscle volumes of the dominant and non-dominant side were confirmed as being normally distributed in both groups. In the comparison of muscle volumes, a two-way ANOVA adjusted for multiple comparisons using the Bonferroni method was used to examine the effect of both the group (golfer vs. non-golfer) and body side (dominant vs. non-dominant) of each trunk muscle. For the comparison of the total muscle volume between the dominant and non-dominant side and the asymmetry ratio between the two groups, an independent t-test was used. All statistical analysis was performed with the IBM Statistical Package for the Social Sciences Statistics for Windows, version 19 (IBM Corp., Armonk, N.Y., USA). The probability level for statistical significance was set at 0.05.

## Results

**Fig 2:**
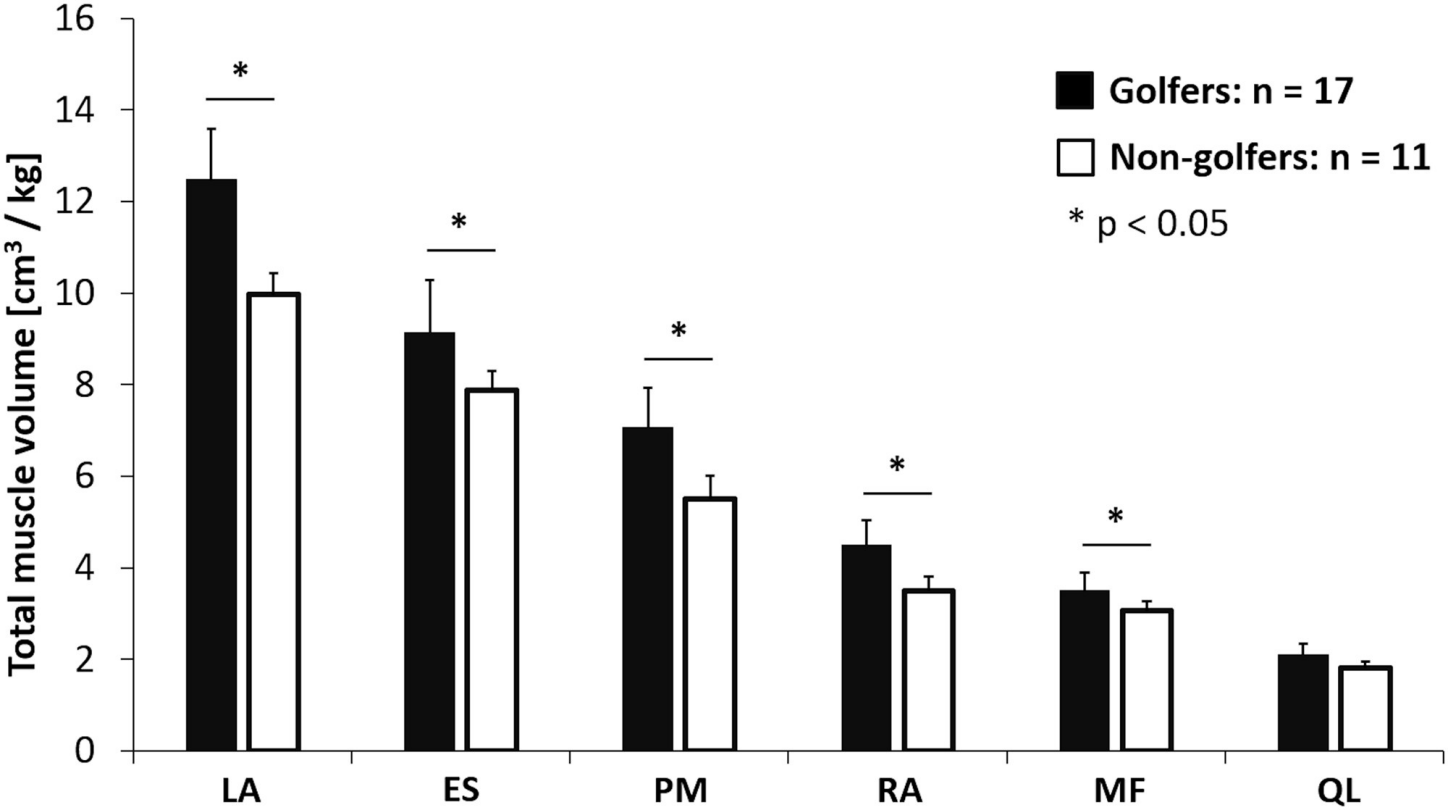
Total muscle volume in golfers and non-golfers after normalizing for body mass. LA, lateral abdominal wall; ES, erector spinae; PM, psoas major; RA, rectus abdominis; MF, multifidus; QL, quadratus lumborum. *, statistically significant difference (*p* < 0.05).

The trunk muscle volume of golfers was significantly larger than that of non-golfers (LA; 12.36 ± 1.12 vs. 9.96 ± 0.94 cm^3^/kg, ES; 9.12 ± 1.16 vs. 7.88 ± 0.84 cm^3^/kg, PM; 6.27 ± 0.88 vs. 5.51 ± 0.98 cm^3^/kg, RA; 4.15 ± 0.54 vs. 3.50 ± 0.64 cm^3^/kg, MF; 3.61 ± 0.41 vs. 3.05 ± 0.40 cm^3^/kg, *p* < 0.05), except for QL muscle volume (1.81 ± 0.24 vs. 1.82 ± 0.25 cm^3^/kg, *p* > 0.05) (Fig 2). Table 2 summarizes the muscle volume on the dominant and non-dominant side and the asymmetry ratio for each trunk muscle in golfers and non-golfers. In the golfers, the muscle volume was significantly larger on the non-dominant side than on the dominant side for the LA, ES, and PM, while the muscle volume on the dominant side was significantly larger than on the non-dominant side for the RA and MF. In non-golfers, there was a significant difference between muscle volumes on the dominant and non-dominant side for the LA and MF.

**Table 2:** Volume of trunk muscles on the dominant and non-dominant side and asymmetry ratio in golfers and non-golfers.

Muscles	Golfers: n = 17			Non-golfers: n = 11		
	Dominant side (cm^3^/kg)	Non-dominant side (cm^3^/kg)	Asymmetry (%)	Dominant side (cm^3^/kg)	Non-dominant side (cm^3^/kg)	Asymmetry (%)
**LA**	5.89 ± 0.55^†^	6.48 ± 0.65*^†^	-8.63 ± 7.40^†^	5.03 ± 0.47	4.93 ± 0.49*	1.94 ± 2.76
**ES**	4.46 ± 0.56	4.65 ± 0.63*	-4.28 ± 6.25	3.91 ± 0.42	3.98 ± 0.44	-1.90 ± 5.64
**PM**	3.00 ± 0.42	3.27 ± 0.47*^†^	-9.10 ± 5.25^†^	2.74 ± 0.47	2.76 ± 0.52	-0.48 ± 5.96
**RA**	2.15 ± 0.32^†^	2.00 ± 0.24*^†^	6.36 ± 6.50^†^	1.74 ± 0.34	1.76 ± 0.31	-2.12 ± 9.64
**MF**	1.84 ± 0.21	1.78 ± 0.21*	3.27 ± 5.37	1.56 ± 0.19	1.50 ± 0.21*	4.15 ± 4.62
**QL**	0.91 ± 0.11	0.90 ± 0.15	0.99 ± 9.03	0.89 ± 0.12	0.93 ± 0.14	-4.56 ± 11.69

Mean ± SD. LA, lateral abdominal wall; ES, erector spinae; PM, psoas major; RA, rectus abdominis; MF, multifidus; QL, quadratus lumborum.

*, significant difference (*p* < 0.05), dominant side vs. non-dominant side;

^†^, significant difference (*p* < 0.05), golfers vs. non-golfers.

The value of the asymmetry ratio of the LA and PM in the golfers was significantly smaller than the value in the non-golfers while the value of the asymmetry ratio of the RA in the golfers was significantly larger than the value in the non-golfers. There was no significant difference in the value of the asymmetric ratio of the ES, MF and QL between golfers and non-golfers.

**Fig 3:**
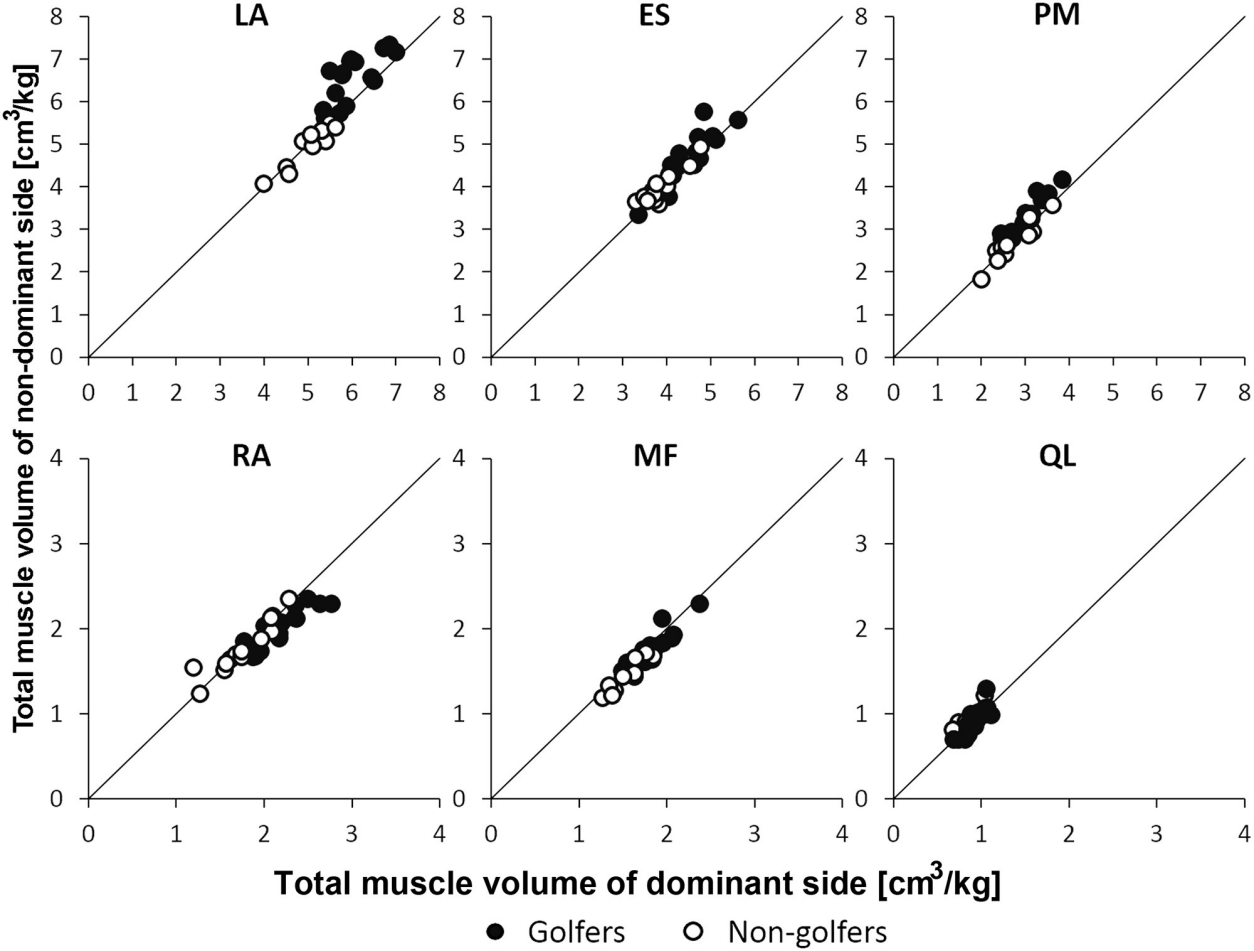
Total muscle volume of the dominant and non-dominant trunk side in golfers and non-golfers. LA, lateral abdominal wall; ES, erector spinae; PM, psoas major; RA, rectus abdominis; MF, multifidus; QL, quadratus lumborum.

Fig 3 shows the distribution of the dominant and non-dominant side trunk muscle volume of each subject. For the LA and PM, most of the golfers’ values are plotted above the line, while most of the non-golfers’ values are plotted nearby or below the line. For the RA, most of the golfers’ values were plotted below the line, while most of non-golfers’ values are distributed on or above the line.

## Discussion

The present study is the first to show the total trunk muscle volume for both the right and left body side and the bilateral differences in these volumes in skilled golfers. The golfers in this study, who performed the game at a relatively high level and had been playing for more than ten years, had a larger trunk muscle volume, except for the QL, than non-golfers, while basic physical characteristics did not differ between the two groups. The primary results of this study were that the golfers had bilateral differences in their trunk muscle volumes (LA, ES, PM, RA, and MF) compared to non-golfers. In particular, the degree of the bilateral difference in the volume of the LA, PM, and RA in the golfers was significantly larger than that of the non-golfers. The golfers had a greater LA and PM volume on the non-dominant compared to the dominant side (meaning the side of their dominant hand), and greater RA volume on the dominant compared to the non-dominant side. The findings of this study confirm our original hypothesis that long-term golf play leads to golf-specific trunk muscle hypertrophy.

Our finding that the RA is bilaterally hypertrophied in golfers is interesting. Previous studies have shown that bilateral asymmetry of trunk muscles occurs in athletes who require trunk rotation in one direction [1, 4, 11]. Tennis players had a 58% larger RA volume compared to non-active people, and RA volume was 35% larger on the non-dominant compared to the dominant side where they hold the racket [1, 4]. This is because trunk lateral flexion to the non-dominant side is affected by the RA of the non-dominant side, which also carries the mechanical load, in tennis players during their serve. From an anatomical standpoint, this appears illogical, since the major role of the RA is forward flexion of the trunk, but an additional role of the RA is lateral flexion of the trunk to the side where it contracts [10]. Right-handed golfers laterally flex their trunk to the dominant (right) side during the downswing [5, 6, 12]. EMG studies support this explanation, since asymmetric activation of the RA muscles was observed during the downswing [7, 8], meaning the RA muscle on the dominant side was activated to a greater extent than the RA on the non-dominant side. Therefore, frequent golf practice could lead to a hypertrophied RA muscle on the dominant (right), but not the non-dominant (left) side, in right-handed golfers.

In this study, the LA showed a large total muscle volume and bilateral differences in golfers. Previous studies demonstrated that tennis players had 30% larger LA volumes compared to non-active people, and the LA had an 18% larger volume on the non-dominant compared to the dominant side [4]. A larger LA on the left side in golfers is most likely associated with trunk motion during a right-handed golf swing. Right-handed golfers rotate their trunk to the left side during the downswing [5, 6, 12]. The major role of the internal oblique is to rotate the trunk to the ipsilateral side. The LA muscles on both sides are activated separately and asymmetrically during the downswing [7, 8]. Thus, the golf swing generates asymmetric activation and, in the long term, hypertrophy of the LA on the non-dominant side. Moreover, in this study, hypertrophy in the LA exceeded the hypertrophy of all other trunk muscles in golfers. The asymmetry ratio of the LA was the second largest of the six examined trunk muscles. In recent studies of baseball players, bilateral asymmetry was observed in the LA muscles, and muscle thickness was positively correlated with bat swing speed [2]. Considering the points above, asymmetric LA hypertrophy might be essential for improving the speed of trunk rotation. However, in order to discuss a potential mechanism for improving the speed of the golf swing, further studies are required to elucidate the effect of asymmetric trunk muscle hypertrophy on club head speed.

In addition, the volume and asymmetry ratio of the PM of golfers was significantly different to that of non-golfers, and the PM on the non-dominant side was significantly larger than that of the dominant side. The major role of the PM is hip flexion, meaning that the PM is one of the major contributors to ipsilateral hip flexion [10]. The PM of elite Australian football players shows bilateral asymmetry of about 3.5% larger cross sectional area on the side of the dominant kicking leg, due to repetitive kicking throughout the season using mainly the same leg [13, 14]. A previous study in right-handed golfers indicated that left hip joint torques were generated in the direction of flexion during the downswing [15]. These results show that the PM on the non-dominant side may be used for rotating the trunk to the non-dominant side during the downswing. In tennis players, the iliacus and psoas muscles, which support similar movements as the PM, show bilateral asymmetry with a 13% greater volume on the non-dominant side [11]. Therefore, recurrent right-handed golf swing practice causes asymmetrical hypertrophy of the left PM, because left hip flexion during the downswing causes a mechanical load on the left PM in right-handed golfers. However, to draw definite conclusions, EMG studies are required to investigate PM activation during the golf swing.

Previous studies analyzed the cross sectional area and volume of several trunk muscles in elite athletes across different sports [1–4, 11, 14, 16–18]. The present study for the first time investigated total muscle volume and bilateral muscle asymmetry in skilled golfers’ trunk muscles. These bilateral differences in trunk muscle size were also observed in players of other sports that require asymmetric trunk motion, such as during throwing and hitting [1–4, 11, 14]. Therefore, we can compare the data of this study with a few studies that determined trunk muscle size and bilateral asymmetry in other asymmetrical sports. It would be beneficial for elite athletes to understand the characteristics and similarities of trunk muscle hypertrophy in asymmetrical sports.

## Conclusions

The results of this study demonstrate that skilled long-term golfers develop large volumes and bilateral asymmetry of their trunk muscles. The LA and PM on the non-dominant side and the RA on the dominant side are required to swing golf clubs, and this results in the volume of these muscles becoming significantly larger than that in non-golfers.

## References

[1] Sanchis-Moysi J, Idoate F, Dorado C, Alayon S, Calbet JA. Large asymmetric hypertrophy of rectus abdominis muscle in professional tennis players. PLoS One. 2010;5: e15858 10.1371/journal.pone.0015858 21209832 PMC3013134

[2] Tsuchikane R, Higuchi T, Suga T, Wachi M, Misaki J, Tanaka D, et al Relationships between bat swing speed and muscle thickness and asymmetry in collegiate baseball players. Sports. 2017;5: 33–40. 10.3390/sports5020033 PMC5968998 29910393

[3] Hides J, Stanton W, Freke M, Wilson S, McMahon S, Richardson C. MRI study of the size, symmetry and function of the trunk muscles among elite cricketers with and without low back pain. Br J Sports Med. 2008;42: 809–813. 10.1136/bjsm.2007.044024 18065440

[4] Sanchis-Moysi J, Idoate F, Izquierdo M, Calbet JA, Dorado C. The hypertrophy of the lateral abdominal wall and quadratus lumborum is sport-specific: an MRI segmental study in professional tennis and soccer players. Sports Biomech. 2013;12: 54–67. 10.1080/14763141.2012.725087 23724609

[5] Horan SA, Evans K, Morris NR, Kavanagh JJ. Thorax and pelvis kinematics during the downswing of male and female skilled golfers. J Biomech. 2010;43: 1456–1462. 10.1016/j.jbiomech.2010.02.005 20185139

[6] Horan SA, Kavanagh JJ. The control of upper body segment speed and velocity during the golf swing. Sports Biomech. 2012;11: 165–174. 10.1080/14763141.2011.638390 22900398

[7] Watkins RG, Uppal GS, Perry J, Pink M, Dinsay JM. Dynamic electromyographic analysis of trunk musculature in professional golfers. Am J Sports Med. 1996;24: 535–538. 10.1177/036354659602400420 8827315

[8] Marta S, Silva L, Vaz J, Bruno P, Pezarat-Correia P. Electromyographic analysis of trunk muscles during the golf swing performed with two different clubs. Int J Sports Sci & Coaching. 2013;8: 779–787. 10.1080/02640414.2015.1069376 26197882

[9] Bulbulian R, Ball KA, Seaman DR. The short golf backswing: effects on performance and spinal health implications. J Manipulative Physiol Ther. 2001;24: 569–575. 10.1067/mmt.2001.118982 11753330

[10] Schunke M, Schulte E, Schumacher U. PROMETHEUS LernAtlas der Anatomie: Allgemeine Anatomie und Bewegungssystem. Auflage 4 In: Rumpfwand. Thieme; 2014 pp. 100–188.

[11] Sanchis-Moysi J, Idoate F, Izquierdo M, Calbet JA, Dorado C. Iliopsoas and gluteal muscles are asymmetric in tennis players but not in soccer players. PLoS One. 2011;6: e22858 10.1371/journal.pone.0022858 21829539 PMC3146492

[12] Okuda I, Gribble P, Armstrong C. Trunk rotation and weight transfer patterns between skilled and low skilled golfers. J Sports Sci Med. 2010;9: 127–133. 24149396 PMC3737954

[13] Stewart S, Stanton W, Wilson S, Hides J. Consistency in size and asymmetry of the psoas major muscle among elite footballers. Br J Sports Med. 2010;44: 1173–1177. 10.1136/bjsm.2009.058909 19474005

[14] Hides J, Fan T, Stanton W, Stanton P, McMahon K, Wilson S. Psoas and quadratus lumborum muscle asymmetry among elite Australian Football League players. Br J Sports Med. 2010;44: 563–567. 10.1136/bjsm.2008.048751 18801772

[15] Foxworth JL, Millar AL, Long BL, Way M, Vellucci MW, Vogler JD. Hip joint torques during the golf swing of young and senior healthy males. J Orthop Sports Phys Ther. 2013;43: 660–665. 10.2519/jospt.2013.4417 23886577

[16] Hoshikawa Y, Muramatsu M, Iida T, Ii N, Nakajima Y, Kanehisa H. Sex differences in the cross-sectional areas of psoas major and thigh muscles in high school track and field athletes and nonathletes. J Physiol Anthropol. 2011;30: 47–53. 21483176 10.2114/jpa2.30.47

[17] Kubo J, Ohta A, Takahashi H, Kukidome T, Funato K. The development of trunk muscles in male wrestlers assessed by magnetic resonance imaging. J Strength Cond Res. 2007;21: 1251–1254. 10.1519/R-19815.1 18076225

[18] Iwai K, Okada T, Nakazato K, Fujimoto H, Yamamoto Y, Nakajima H. Sport-specific characteristics of trunk muscles in collegiate wrestlers and judokas. J Strength Cond Res. 2008;22: 350–358. 10.1519/JSC.0b013e3181635d25 18550947

